# Diagnostic Advancements in MINOCA: Do They Translate to a Better Clinical Outcome? A Review of the Literature

**DOI:** 10.3390/medicina62071243

**Published:** 2026-06-27

**Authors:** Maria Bozika, Anastasios Apostolos, Kassiani-Maria Nastouli, Georgios Boliaris, Athanasios Sakalidis, Nikolaos Ktenopoulos, Paschalis Karakasis, Ioannis Skalidis, Konstantinos Konstantinou, Emmanouil Mantzouranis, Ioannis Leontsinis, Grigorios Tsigkas, Kyriakos Dimitriadis, Konstantinos Tsioufis, Vasileios Panoulas

**Affiliations:** 1 Department of Medicine, Division of Cardiology, University Hospital of Patras, 26504 Patras, Greece; mariabozika29@gmail.com (M.B.); kassienmarie@gmail.com (K.-M.N.); gmpoliaris@gmail.com (G.B.); gregtsig@upatras.gr (G.T.); 2 Department of Cardiology, Royal Brompton and Harefield Hospitals, Guy’s and St Thomas’ NHS Foundation Trust, London UB9 6JH, UK; asakalidis@gmail.com (A.S.); vasileios.panoulas2@nhs.net (V.P.); 3 Faculty of Medicine, Imperial College London, London SW7 2AZ, UK; 4 First Department of Cardiology, Medical School, General Hospital of Athens Hippocration, National and Kapodistrian University of Athens, 11527 Athens, Greece; nikosktenop@gmail.com (N.K.); kostiskon@gmail.com (K.K.); giannisleontsinis@gmail.com (I.L.); dimitriadiskyr@yahoo.gr (K.D.); ktsioufis@gmail.com (K.T.); 5 Second Department of Cardiology, Hippokration General Hospital, Aristotle University of Thessaloniki, 54124 Thessaloniki, Greece; pakar15@hotmail.com; 6 Department of Cardiology, HFR—Fribourg Cantonal Hospital and University, 1708 Fribourg, Switzerland; 7 London Health Science Centre, Western University, London, ON N6A 5W9, Canada; mantzoup@gmail.com

**Keywords:** MINOCA, cardiac magnetic resonance, optical coherence tomography, intravascular ultrasound, coronary functional testing, acetylcholine provocation, secondary prevention

## Abstract

Myocardial infarction with non-obstructive coronary arteries (MINOCA) accounts for approximately 5–15% of all myocardial infarctions and disproportionately affects women. Once treated as a diagnosis of exclusion, MINOCA is now recognised as a heterogeneous, mechanism-based syndrome encompassing atherosclerotic plaque disruption, epicardial and microvascular vasospasm, microvascular dysfunction, coronary thromboembolism, and spontaneous coronary artery dissection (SCAD). Despite the absence of obstructive disease, it carries substantial morbidity and mortality, underscoring the need for accurate aetiological characterisation and tailored therapy. Our aim is to review the contemporary evidence of the role of advanced imaging modalities—cardiac magnetic resonance imaging (CMR), optical coherence tomography (OCT), intravascular ultrasound (IVUS) and invasive functional testing—in the diagnosis, prognostic stratification, and therapeutic guidance of patients with MINOCA. CMR is the non-invasive reference standard for differentiating true ischaemic MINOCA from non-ischaemic mimics such as myocarditis and Takotsubo syndrome, reclassifying the working diagnosis in up to two-thirds of cases. OCT and IVUS provide intracoronary characterisation of culprit substrates that are invisible via angiography, particularly plaque rupture, erosion, intramural haematoma and SCAD, while acetylcholine and adenosine testing identify endothelium-dependent vasospasm and endothelium-independent microvascular dysfunction respectively. Coronary Computed Tomography Angiography (CCTA) could also play an additional role in the diagnosis of epicardial CAD. Each modality additionally carries independent prognostic value, with abnormal findings consistently linked to higher rates of major adverse cardiovascular events. The recently completed PROMISE trial provided the first randomised evidence that stratified, imaging-guided treatment might have some positive impact on angina status and quality of life compared with empirical standard care. In conclusion, advanced imaging has transformed MINOCA from a diagnosis of exclusion into a mechanism-based syndrome amenable to personalised therapy. Broader integration of these modalities into routine practice, supported by further randomised trials, is needed to optimise outcomes.

## 1. Introduction

Myocardial infarction with non-obstructive coronary arteries (MINOCA) is a well-known term used to describe patients who meet the criteria for myocardial infarction (MI) according to the fourth universal definition but for whom a coronary angiogram does not show any obstructive coronary artery disease (≥50% stenosis). Additionally, neither alternative non-cardiac causes of MINOCA like sepsis or pulmonary embolism, nor cardiac causes like myocarditis, tachycardiomyopathy or Takotsubo syndrome, explain its acute presentation [1,2]. Reported prevalences fall in the 5–15% range for all MIs presenting with non-significant obstruction [2], while this figure additionally represents the ratio of all MI patients undergoing cardiac catheterization [1]. MINOCA also appears to disproportionately affect women [3,4,5,6]. Historically considered a diagnosis of exclusion, mainly due to the inadequate detection of its pathogenesis, a MINOCA diagnosis was based solely upon the elimination of alternative MI causes. In recent years, however, while the same exclusion criterion applies, advanced diagnostic techniques such as CMR, OCT, and invasive functional testing are proving increasingly successful in identifying the aetiology of MINOCA [1,7,8]. Having been shown to be far from a benign clinical entity, causing significant mortality and morbidity [9], along with anxiety and depression [5,10], the need for optimized detection, risk stratification and personalized treatment renders evaluation of the relevant literature necessary. The aim of our review is to summarize the recent evidence on diagnostic advancements in MINOCA and update the literature in comparison with existing reviews.

## 2. Methods

This review is a narrative review rather than a systematic review or meta-analysis. Accordingly, no formal risk-of-bias assessment (e.g., ROBINS-I or Cochrane RoB tool) was applied to included studies, and no quantitative data pooling was performed. A structured search strategy and thematic synthesis were employed to ensure comprehensiveness and transparency rather than to meet the criteria of a formal systematic review.

### 2.1. Information Sources and Search Strategy

A literature search was performed in PubMed/MEDLINE, Embase, the Cochrane Central Register of Controlled Trials, and Google Scholar from database inception through March 2026. The following terms were combined using Boolean operators: “MINOCA”, “myocardial infarction with non-obstructive coronary arteries”, “myocardial infarction non-obstructive”, “cardiac magnetic resonance”, “CMR”, “late gadolinium enhancement”, “optical coherence tomography”, “OCT”, “intravascular ultrasound”, “IVUS”, “acetylcholine provocation”, “coronary functional testing”, “coronary microvascular dysfunction”, “coronary vasospasm”, “plaque erosion”, “plaque rupture”, “Coronary Computed Tomography Angiography” and “spontaneous coronary artery dissection”. The reference lists of all included articles, of major scientific statements, and of recent narrative and systematic reviews were hand-searched to identify additional relevant publications.

### 2.2. Inclusion Criteria

(i) Studies published in English language in peer-reviewed journals; (ii) original research (randomised controlled trials, prospective and retrospective observational cohorts, and registry analyses), systematic reviews, meta-analyses, and authoritative scientific statements or guidelines issued by recognised cardiovascular societies; (iii) populations conforming to the contemporary working definition of MINOCA, i.e., fulfilment of the Fourth Universal Definition of Myocardial Infarction with <50% angiographic stenosis and exclusion of clinically overt non-ischaemic alternative diagnoses; and (iv) studies addressing the diagnostic performance, pathophysiological characterisation, prognostic value, or therapeutic implications of CMR, OCT, IVUS, or invasive functional testing in MINOCA.

### 2.3. Exclusion Criteria

(i) Isolated case reports and small case series (<10 patients), unless of unique mechanistic value; (ii) editorials, commentaries and correspondence without primary data; (iii) studies of obstructive coronary artery disease in which MINOCA-relevant findings could not be separately extracted; and (iv) non-English publications without an available translation.

### 2.4. Synthesis

Selected studies were appraised for relevance, methodological robustness, and applicability to contemporary practice. As this is a narrative rather than systematic review, no formal quantitative pooling was performed; quantitative findings from individual studies and from previously published meta-analyses are cited as originally reported. Evidence is organised thematically into categories encompassing pathophysiology and heterogeneity, diagnostic modalities, post-imaging diagnostic reclassification, prognostic implications, therapeutic considerations, current limitations of imaging-guided care, and future directions.

## 3. Pathophysiology and Heterogeneity

MINOCA has been termed an umbrella diagnosis due to its heterogenous aetiology. Coronary plaque disruption seems to be a common culprit lesion causing MINOCA, encompassing plaque rupture, plaque erosion, intra-plaque haemorrhage, layered plaque and calcific nodules [1,2,5,6,11], while it is specifically found to be the most common cause in women [4,12]. The myocardial damage in this case stems from thrombus formation, causing distal embolization, secondary vasospasm or local coronary thrombosis, significantly occluding the lumen, followed by spontaneous resolution, all of which result in cellular necrosis without apparent vessel occlusion [2,5,11,13,14]. Another cause of MINOCA is coronary vasospasm (>90% occlusion), which can be exogenously initiated (i.e., drug- or toxin-induced) or result from defective smooth muscular wall dysfunction. It is of note that vasospastic angina is more common in patients with nonobstructive coronaries. Microvascular dysfunction is another causal mechanism of MINOCA, as it is undetectable by conventional coronary angiography, even though microcirculation accounts for about 70% of coronary resistance in the absence of obstructive coronary artery disease (CAD) [2,15]. Such cases are further subcategorized into endothelium-dependent and endothelium-independent (smooth muscle-induced) cases, a distinction that can be established via the imaging techniques discussed below [15]. Additionally, coronary thrombosis or embolism can result in MINOCA in cases in which the occlusions affect the microcirculation or are thrombolyzed enough by the time of the angiography that the blockage is not classified as clinically significant, situations that may or may not involve hypercoagulability. Spontaneous Coronary Artery Dissection (SCAD) is a rare causative agent of MINOCA whereby the separation of the tunica media and adventitia obstructs flow due to the intramural haematoma protruding into the lumen (although whether the primary plane of dissection is intimal or medial remains debated). A supply–demand mismatch could present as MINOCA, both clinically and imaging-wise, although in the presence of a reasonable cause of the mismatch (i.e., hypotension, tachyarrhythmia, or anaemia), a diagnosis of MI type-2 should be made [2]. Thus, it is evident that MINOCA results from a spectrum of pathogeneses, underlining the importance of novel imaging modalities in higher-specificity diagnosis.

## 4. Diagnostic Advancements

### 4.1. Cardiac Magnetic Resonance Imaging

**Figure 1:**
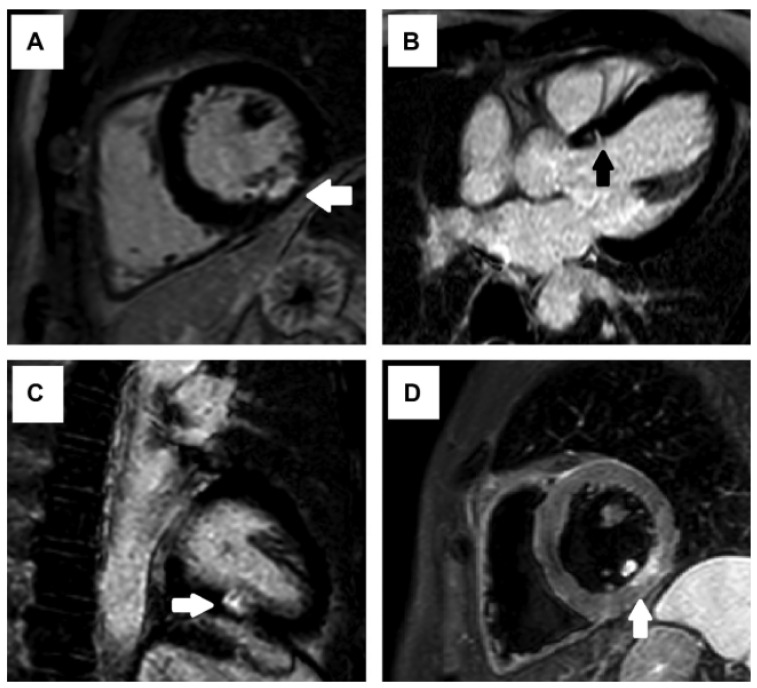
Cardiac magnetic resonance. (**A**,**C**) LGE: Almost transmural lesions in mid- and apical inferolateral segments and the posteromedial papillary muscle. (**B**) LGE: Another focal subendocardial lesion in the basal anteroseptal segment of the left ventricle. (**D**) STIR-T2: Oedema in the mid-inferolateral segment of the left ventricle, as well as in the posteromedial papillary muscle. LGE, late gadolinium enhancement; STIR-T2, short TI inversion recovery.

Cardiac magnetic resonance imaging uses magnetic fields and radiofrequency pulses to generate a high-resolution assessment of regional wall-motion abnormalities and allows for accurate quantification of ventricular function. Its principal sequences are T2-weighted imaging, which detects oedema; T1-weighted parametric mapping; and late gadolinium enhancement (LGE), which identifies inflammation-related injuries, necrosis, scarring and infiltration [12,16,17]. Myocardial oedema, as evidenced by T1 and T2 hyperintensity, indicates the site of acute injury (particularly indicative of coronary stemmed ischaemia if the affected area matches the distribution of a vessel), whereas LGE is essential for differentiating acute ischaemic injury from non-ischaemic aetiologies [12]. Specifically, intramyocardial or subepicardial LGE patterns are suggestive of a non-ischaemic process—most commonly, myocarditis—while subendocardial (partial-thickness) or transmural (full-thickness) enhancement is characteristic of ischaemic injury [4]. Owing to its ability to characterize both reversible injury (such as inflammation and oedema) and irreversible damage (such as necrosis and fibrosis), CMR is considered the gold-standard non-invasive imaging modality for the diagnosis of MINOCA [5,18]. Furthermore, CMR helps distinguish Takotsubo cardiomyopathy due to the absence of LGE, uniform regional distribution of oedema, and specific wall-motion abnormalities characterized by ballooning at various ventricular levels. Despite its contribution to the visualisation of myocardial status due to its ability in tissue characterization, CMR identifies culprit vascular lesions less accurately than OCT [5]. Interestingly, in a systematic review and meta-analysis of patients with a working diagnosis of MINOCA receiving CMR within 10 days of the acute event, this imaging modality achieved definite diagnosis in 63% of patients, leading to a major reclassification of diagnosis and differentiating MINOCA from non-ischaemic conditions. Major Adverse Cardiovascular Events (MACEs) were found to mainly occur in the CMR-diagnosed MINOCA group (pooled OR: 2.40 [95% CI: 1.60–3.59]), as opposed to the non-ischaemic aetiologies of myocarditis (pooled OR: 1.09 [95% CI: 0.41–2.91]) and Takotsubo syndrome (pooled OR: 1.16 [95% CI: 0.62–2.16]) [19], showcasing the prognostic utility of CMR (Figure 1). The critical question, however, is whether CMR’s diagnostic yield translates into meaningful improvements in patient outcomes. The available evidence suggests an indirect but clinically important link. By accurately reclassifying up to two-thirds of working MINOCA diagnoses—redirecting patients with myocarditis or Takotsubo syndrome away from antiplatelet and statin regimens they do not need and confirming true ischaemic MINOCA in those who do—CMR enables mechanism-appropriate therapy allocation that is biologically expected to improve outcomes. The prognostic data support this reasoning, as we mentioned earlier. This prognostic stratification has direct therapeutic consequences. Nevertheless, it remains important to acknowledge that no randomised trial has yet demonstrated that CMR-guided treatment allocation reduces hard cardiovascular events in MINOCA; the outcome link at this stage is observational and mechanistically inferred rather than proven by RCT-level evidence.

### 4.2. Optical Coherence Tomography

OCT uses near-infrared light delivered via an intracoronary catheter to create high-resolution cross-sectional images of the coronary artery wall and lumen, allowing for assessment of their microstructure in detail, including their plaque morphology (fibrous cap and lipid core), thrombus, and plaque rupture/erosion [20]. It can distinguish between the different components of plaque and thrombus by utilizing the unique optical properties of each tissue, such as fibrous, lipidic, and calcified components [11]. Additionally, OCT can detect coronary spasm, viewed as intimal bumping [5], or wavy intima compressed by the contracted media [12,21]. The study reporting the latter phenomenon attributed its low incidence during OCT to the nitroglycerine typically given before OCT, along with anticoagulation, pointing out that spasm may be more common than originally thought. Remarkably, although an OCT-detected culprit lesion was less frequent in vessels with angiographically normal findings, the likelihood of identifying a culprit lesion in vessels with nonobstructive disease was not significantly associated with stenosis severity in women. This is consistent with the finding that the culprit lesion is not usually located at the point of maximal stenosis, suggesting that OCT performed solely on the basis of angiographic findings may not yield the best chances of discovering the primary lesion. It is recommended that patients with smaller increases in troponin not be dismissed, as they are as likely to have OCT-determined lesions as those with larger increases [12]. Moreover, due to its ability to distinguish structural plaque characteristics, OCT has been used to differentiate the mechanism of MI between MINOCA and CAD-MI in women: ruptured plaque and intraplaque haemorrhage were more prevalent in MINOCA patients, while plaque rupture with a persistent cavity and thrombus were mainly found in obstructed vascular lesions [11]. One OCT study states that non-atherosclerotic causes of MINOCA are more prevalent in women, while atherosclerotic MINOCA is more common in men [6]. A different study including both sexes reported 80% sensitivity upon aetiological detection of MINOCA, mentioning that a thrombus was found in all ruptured plaques of the participants’ vessels [22]. Lastly, it has been demonstrated that the combination of OCT with CMR yields a higher MINOCA identification rate, with a reported 85% of patients being diagnosed in the HARP-MINOCA female-only study [12], while all patients were diagnosed or had a substrate identified in Gerbaud et al. cohort including both sexes [22]. It is of note that, while CMR is currently considered the gold standard for MINOCA diagnosis, it falls behind on the ability to aetiologically classify the index ACS event compared to OCT, owing to the latter’s intraluminal and wall-viewing ability. Whether OCT’s diagnostic precision translates into better outcomes remains an evolving question. The indirect evidence is promising: in the Gerbaud et al. cohort, OCT changed clinical management in 27.5% of patients—nearly double the rate attributed to CMR alone—suggesting that OCT-guided therapy allocation meaningfully redirects treatment [22]. At the prognostic level, atherosclerotic MINOCA identified by OCT is associated with substantially higher MACE rates than non-atherosclerotic MINOCA (15.3% vs. 4.5%, *p* = 0.015) and greater target-lesion revascularisation (6.1% vs. 0%, *p* = 0.030), confirming that OCT-derived phenotyping identifies a genuinely higher-risk subgroup warranting more intensive secondary prevention. Taruya et al. further demonstrated that OCT-detected high-risk lesions predict the site of future ACS with spatial precision, supporting the case for targeted treatment of OCT-identified substrates [9]. However, whether systematically applying OCT-guided management actually reduces subsequent MACE has not been explored in a dedicated randomised trial in MINOCA. The outcome benefit of OCT at this stage remains biologically reasonable and observationally supported but not yet proven.

### 4.3. Intravascular Ultrasound

Intravascular ultrasound (IVUS) utilizes a catheter-mounted, high-frequency ultrasound transducer to generate real-time, cross-sectional images of the vessel wall by emitting and receiving acoustic signals from within the coronary artery. Its use cases include the detection of plaque rupture; ulceration/erosion; thickness; and the presence/absence of thrombus, dissection, and calcification, as well as the classification of the plaque type as soft, calcified, fibrous, or mixed. Interestingly, advances in adjunctive techniques currently used primarily in research, such as near-infrared spectroscopy (NIRS) combined with IVUS, enable the characterization of lipid content within the arterial wall, thereby providing insight into plaque vulnerability [5,23]. Given that culprit plaques in MINOCA, particularly those with ulceration, often exhibit substantial lipid components, the clinical adoption of this technology is likely to significantly enable the evaluation of MINOCA patients. Despite OCT providing higher resolution and detail endoluminally, IVUS offers deeper tissue penetration, thereby providing information about deeper plaque layers, and does not require contrast, making it preferable to OCT for patients with chronic kidney disease [5,15]. In a female-only cohort, patients with plaque disruption had a higher level of average maximal stenosis in angiography, and although plaque ruptures were identified via IVUS, even in normal-appearing segments, in patients with completely normal angiograms, no plaque ruptures were detected. There was no relationship between plaque disruption and ECG or wall-motion changes. Lastly, CMR was found to offer information complementary to IVUS, achieving 70% combined detection of abnormalities in this MINOCA cohort [4]. The outcome implications of IVUS in MINOCA are currently the least characterised of the major imaging modalities, and it is important to state this limitation plainly. While IVUS identifies plaque features—rupture, lipid burden, and non-obstructive plaque in angiographically normal segments—that carry prognostic relevance in the broader non-obstructive CAD literature, dedicated outcome studies in MINOCA populations are sparse. IVUS currently contributes most directly to outcomes by informing the therapeutic decision to initiate or withhold antiplatelet and statin therapy in patients with confirmed plaque burden and by identifying SCAD in segments that angiography cannot resolve—decisions with known downstream prognostic consequences. Nevertheless, no randomised or prospective outcome study has evaluated an IVUS-guided treatment strategy specifically in MINOCA, and the outcome contribution of IVUS remains inferred from its diagnostic role rather than having been demonstrated by dedicated clinical-trial evidence.

### 4.4. Functional Testing

Functional testing in MINOCA uncovers mechanisms not visible via angiography, with particular emphasis on vasoreactivity testing using acetylcholine (ACh) and, to a lesser extent, adenosine. ACh testing evaluates endothelium-dependent function: in healthy vessels, it induces vasodilation via nitric oxide (NO) release, whereas in endothelial dysfunction, where NO is missing, it exerts a direct muscarinic effect, causing vasoconstriction. During intracoronary ACh infusion, reproduction of symptoms and ischaemic ECG changes without ≥90% epicardial narrowing indicate microvascular spasm, while the same findings with ≥90% constriction indicate epicardial vasospasm [24]. In contrast, adenosine testing assesses endothelium-independent function, producing direct smooth muscle vasodilation to measure coronary flow reserve (CFR) and the index of microvascular resistance (IMR) [2]. Reduced CFR and/or elevated IMR reflect structural or functional microvascular dysfunction rather than vasospastic reactivity. Thus, ACh and adenosine are complementary: the former reveals vasomotor reactivity, and the latter quantifies flow limitation and resistance. This combined functional approach is highly effective, particularly when integrated with imaging modalities like OCT and IVUS to exclude plaque disruption. It significantly enables diagnosis compared to angiography alone and thereby facilitates mechanism-based treatment. Trials such as CorMicA [25] demonstrate that stratified therapy guided by invasive functional testing improves angina and quality of life, supporting its clinical utility, even if hard outcome data in MINOCA remain limited [15]. Subgroup differences are worth mentioning; women show a high prevalence of coronary microvascular dysfunction, with abnormal CFR observed in up to 47%, making microvascular testing particularly important [26]. Similarly, patients with cardiometabolic risk factors (e.g., diabetes) may exhibit structural microvascular remodelling, which is better captured by IMR than by vasospasm testing alone. Functionally abnormal findings (low CFR, high IMR, or positive ACh test) are also associated with worse prognosis and recurrent symptoms [15]. In addition, the combination of functional assessment and intravascular imaging might be the future [27]. Overall, functional testing in MINOCA is pivotal because it distinguishes endothelium-dependent vasospasm from endothelium-independent microvascular dysfunction, allowing for precise physiology-based management, though further trials are needed to standardize its routine use (Table 1). The outcome translation of this diagnostic precision is only partially established. A positive ACh provocation test independently predicts MACE (HR 2.83, 95% CI: 1.16–6.93; *p* = 0.022) [24], and impaired coronary vasomotor function in MINOCA is associated with higher rates of all-cause mortality, cardiac death, and ACS readmission [15]—confirming that functional testing stratifies patients into meaningfully different risk strata. The CorMicA trial, though not restricted to MINOCA, demonstrated that stratified therapy guided by invasive functional assessment improves angina and quality of life compared with standard care [25], providing proof of concept that physiology-guided management generates patient-relevant benefit. However, whether treatment guided by functional testing reduces hard cardiovascular events specifically in MINOCA remains to be demonstrated in a dedicated randomised trial; current outcome evidence remains observational. Further trials are needed to standardise protocols and establish a definitive causal link between therapy guided by functional testing and improved clinical outcomes in this population.

### 4.5. Coronary Computed Tomography Angiography (CCTA)

Coronary computed tomography angiography (CCTA) occupies a distinct and complementary role in MINOCA’s diagnostic landscape. Unlike OCT and IVUS, which require invasive catheter delivery and are therefore performed at the time of angiography, CCTA is non-invasive and can be deployed either as a gatekeeper investigation in patients who did not undergo invasive coronary angiography at the time of the index event or as a follow-up tool for anatomical and plaque reassessment. Its spatial resolution—while inferior to OCT for intraluminal detail—is sufficient to characterise a range of substrates relevant to MINOCA aetiology that angiography alone cannot resolve.

Plaque characterisation is among CCTA’s most clinically relevant contributions in this context. High-risk plaque features detectable by CCTA—including low-attenuation plaque (LAP), the napkin-ring sign, positive remodelling, and spotty calcification—identify lesions with a thin fibrous cap and large lipid core, the same substrates that OCT demonstrates as plaque rupture or erosion in MINOCA patients [28]. In patients presenting with MINOCA in whom invasive imaging was not performed, CCTA identification of high-risk plaque features in a non-obstructive lesion provides indirect evidence of a plaque disruption aetiology and a rationale for antiplatelet and statin therapy, even in the absence of haemodynamically significant stenosis.

CCTA is also the most sensitive non-invasive modality for the detection of spontaneous coronary artery dissection (SCAD) and coronary anomalies. While SCAD is definitively characterised by OCT or IVUS during the index catheterisation, CCTA can identify double lumen, intramural haematoma, or luminal compression that may be missed on angiography in patients with subtle or partially resolved dissections and can delineate anomalous coronary origins—including interarterial and intramyocardial courses—that may precipitate ischaemia through compression or spasm rather than atherosclerosis [29].

An emerging application of CCTA in MINOCA is the assessment of pericoronary adipose tissue (PCAT) attenuation. PCAT attenuation, measured as the mean CT attenuation of adipose tissue within 3 mm of the outer coronary wall, reflects pericoronary inflammation driven by local vascular pathology. Elevated PCAT attenuation around a non-obstructive segment has been associated with plaque vulnerability and adverse cardiovascular outcomes in broader CAD populations [30], and its application to MINOCA—where pericoronary inflammation may reflect subclinical plaque activity invisible to angiography—represents a promising but, as yet, unvalidated research avenue.

The limitations of CCTA in MINOCA are important to acknowledge. Radiation exposure and the requirement for iodinated contrast limit its use in young patients and those with renal impairment, populations that overlap substantially with MINOCA demographics. Temporal separation from the acute event may result in the resolution of transient findings such as intramural haematoma. Spatial resolution remains insufficient for definitive characterisation of fibrous cap integrity or thrombus composition, tasks for which OCT retains superiority. Finally, dedicated outcome data evaluating CCTA-guided management specifically in MINOCA are absent; its role currently rests on mechanistic plausibility and extrapolation from broader non-obstructive CAD literature rather than trial-level evidence.

## 5. Reclassification of Diagnosis Post CMR

A systematic review and meta-analysis on CMR in MINOCA patients warrants particular attention, with the majority of MINOCA working diagnoses reclassified post imaging. More specifically, 63% of all patients having undergone CMR received a definite diagnosis (95% CI: 0.58–0.66; I^2^ = 95.6%). Of those, 68% received a diagnosis other than MINOCA, highlighting, in practice, why it should be considered a working diagnosis. As previously stated, myocardial oedema indicates the site of acute injury, whereas LGE is essential for the differentiation of acute ischaemic injury from non-ischaemic aetiologies: intramyocardial or subepicardial LGE patterns are indicative of a non-ischaemic process—most commonly, myocarditis—while subendocardial (partial-thickness) or transmural (full-thickness) enhancement is characteristic of ischaemic injury. Thus, CMR was able to detect true ischaemia, i.e., MINOCA, in 22% of the cases, while 31% were classified as myocarditis, 10% as Takotsubo, and 27% had normal findings. The remaining 10% were non-diagnostic or had cardiomyopathies. This major reclassification rate was attributed to the early (within 10 days) performance of the CMR modality, minimizing the chances of findings’ normalization post index event, especially due to the transient nature of oedema being detected on T2WI, as well as to the usage of more novel CMR techniques like parametric mapping. Additionally, epicardial or microvascular causes of true MINOCA being already detected via coronary angiogram or functional testing before patients who had already undergone CMR contributes to the explanation as to why initial MINOCA diagnoses were altered at such a high rate [19]. Therefore, it would be wise to deduce that CMR constitutes a powerful tool in tissue-based assessment of possible MINOCA patients, especially those in whom invasive imaging has failed to establish a definite diagnosis.

## 6. Prognostic Value

Beyond their diagnostic utility, these novel imaging modalities should offer insight into patients’ prognosis and, thus, allow for risk stratification, a parameter described in the literature. Major Adverse Cardiovascular Events (MACEs) can be used as a useful prognostic criterion for patient outcome. In a systematic review and meta-analysis, CMR-confirmed diagnosis of MINOCA was associated with a significantly higher risk of major adverse cardiovascular events (pooled OR: 2.40 [95% CI: 1.60–3.59]). In contrast, diagnoses of myocarditis (pooled OR: 1.09 [95% CI: 0.41–2.91]) and Takotsubo syndrome (pooled OR: 1.16 [95% CI: 0.62–2.16]) were not linked to a significant increase in combined clinical outcomes; data were extracted from different studies with a combined prevalence of 39% for hypertension, 27% for dyslipidaemia, 15% for diabetes, and 23% for smoking. Thus, an ischaemic LGE pattern was found to be mainly associated with MACE, compared to non-ischaemic patterns and normal findings, the latter of which had the best prognosis [19]. In a study following the outcomes of patients initially under a working diagnosis of MINOCA who underwent CMR, true ischaemic aetiology was confirmed in only 25% of the patients, with the rest being reclassified as myocarditis (13%), Non-Ischaemic Cardiomyopathy (NICM) (44%), normal CMR (15%) and other diagnoses (3%). MACE and mortality were assessed in 10 years: true MI carried a 47% chance of MACE and 37% chance of mortality; myocarditis carried 17% and 24% chances, respectively; NICM posed a 50% risk of MACE and 37% for mortality; and normal CMR findings corresponded to a 3.5% chance for each of the two parameters [17].

OCT and IVUS, on the other hand, offer insight into the pathophysiology of the acute index cardiac episode, thereby stratifying patients with the phenotype of plaque disruption into a specific risk quota and opening the door to mechanism-specific treatment. Atherosclerotic plaque rupture and related local or systemic inflammation are associated with an increased risk of recurrent events compared with plaques with an intact fibrous cap or lack of objective inflammation, as viewed intravascularly. In a review of OCT utility in MINOCA, compared to patients with nonatherosclerotic causes, those with atherosclerotic causes of MINOCA had worse clinical outcomes, a higher incidence of MACE (15.3% vs. 4.5%, *p* = 0.015), and more common target-lesion revascularization (6.1% vs. 0%, *p* = 0.030) [6]. In an observational study, Noguchi et al. demonstrated that plaque burden in a non-obstructive left main coronary artery (LMCA), as assessed by IVUS, was independently associated with increased long-term all-cause and cardiac mortality in patients who did not undergo LMCA revascularization, even when the lumen area remained preserved [31]. Taruya et al. examined the prognostic value of OCT in MINOCA. Their study included 82 patients presenting with MINOCA; OCT identified 42 previously unrecognized high-risk lesions (51.2%). Over a 2-year follow-up, 4 of these 42 patients (10%) experienced recurrent ACS, with the culprit lesion located in the same segment as the initially detected high-risk lesion [9]. Zeng et al. studied 190 MINOCA patients using OCT, distinguishing between atheromatic causes of the index episode (33.7%) and non-atheromatic causes (47.9%). They discovered that atheromatic MINOCA patients exhibited worse clinical outcomes, with a higher incidence of MACE (15.3% vs. 4.5%; *p* = 0.015), more frequent target-lesion revascularization (TLRs) (6.1% vs. 0%; *p* = 0.030) and more rehospitalizations for angina (6.1% vs. 0%; *p* = 0.030) [6].

Functional testing also has been assessed as to its prognostic capabilities. Specifically, a study evaluating the safety and complications of ACh testing in MINOCA patients found that patients with a positive ACh provocation test had a higher incidence of MACCE compared to those with a negative test (24 [13.0%] vs. 6 [4.5%]; *p* = 0.017), primarily driven by an increased rate of hospitalization for unstable angina (16 [8.6%] vs. 4 [3.0%]; *p* = 0.049). There were no significant differences between the groups in terms of cardiovascular death (1 [0.5%] vs. 0 [0.0%]; *p* = 0.397), non-fatal myocardial infarction (5 [2.7%] vs. 1 [0.8%]; *p* = 0.226), or cerebrovascular events (3 [1.6%] vs. 1 [0.8%]; *p* = 0.520); however, we should note the small sample size. Additionally, recurrent angina was more frequent in patients with a positive test (58 [31.4%] vs. 14 [10.6%]; *p* < 0.001), and their Seattle Angina Questionnaire (SAQ) summary score at 12 months was lower compared to those with a negative result (82 [IQR 75.5–88] vs. 84 [IQR 78–88]; *p* = 0.022). A positive ACh test (hazard ratio [HR]: 2.83; 95% CI: 1.16–6.93; *p* = 0.022) was also found to be an independent predictor for the occurrence of MACE, along with MINOCA presentation and left ventricular ejection fraction [24]. Mangiacapra et al. stated that although a positive provocative test appeared to only impact patients with unstable angina and non-obstructive coronary arteries, its prognostic significance in patients with acute myocardial infarction is meaningful. In patients with MINOCA, impaired coronary vasomotor function is associated with higher rates of all-cause mortality, cardiac death, and readmission for acute coronary syndromes, along with poorer angina status [15]. These findings support the notion that a positive provocative test in the MINOCA population identifies a higher-risk subgroup.

Although patients with MINOCA generally have a more favourable prognosis than those with MI due to obstructive coronary artery disease (MI-CAD), their risk of major adverse cardiovascular events remains higher than that of the general population. In a systematic review by Pasupathy et al., 12-month all-cause mortality was lower in MINOCA compared with MI-CAD (4.7% vs. 6.7%) [32]. In contrast, Choo et al. reported similar 2-year all-cause mortality between the two groups (9.1% vs. 8.8%) [33]. Despite the absence of obstructive coronary disease, these mortality rates remain substantial, underscoring the clinical importance of identifying and managing MINOCA. Furthermore, in a cohort of 4793 STEMI patients, Andersson et al. demonstrated that individuals with non-obstructive or angiographically normal coronary arteries had comparable or even higher long-term mortality risk than those with obstructive disease. Additionally, deaths in patients without obstructive coronary artery disease were less frequently attributable to cardiovascular causes [34]. Similarly, in the SWEDEHEART study (mean follow up of 4.1 years), mortality was 13.4%; 7.1% of patients experienced another myocardial infarction, 4.3% had ischaemic stroke, 6.4% were hospitalized for heart failure, and hospitalization for bleeding occurred in 3.6%, with less than half of all deaths classified as cardiovascular. One-year mortality in young patients with MINOCA is reportedly lower (1.7%). Approximately 25% of patients with MINOCA will experience angina in the subsequent 12 months, which is similar to the frequency reported in patients with AMI-CAD [35]. Therefore, the overall high mortality and morbidity associated with MINOCA necessitate personalized risk stratification and mechanism-based treatment, parameters that novel imaging modalities can contribute to.

## 7. Clinical Impact and Therapeutic Implications

MINOCA lacks significant evidence-based literature and prospective randomized and controlled trials, making its optimal management less clear compared to AMI-CAD. Emergent supportive care; a working diagnosis approach; cardioprotective therapies, irrespective of the cause; and cause-targeted therapies are all recommended in MINOCA cases. The role novel diagnostic imaging modalities can play in this chain of interventions is worth exploring. More specifically, although emergent pathologies like arrhythmias can be addressed without the use of imaging, the subsequent discovery of the mechanism of their induction— for example, ventricular arrhythmia resulting from persistent spasm discovered by OCT—can allow for treatment of the root cause. As we mentioned before, MINOCA should constitute a working diagnosis, with the clinician trying to rule out non-ischaemic MINOCA mimics like Takotsubo syndrome or myocarditis, in addition to uncovering the mechanism of the acute episode [2,17]. Both these purposes can be enabled by the use of CMR and/or invasive imaging; it has been shown that CMR, especially in combination with OCT, can distinguish MINOCA from non-ischaemic process diagnosis [6,17,19] and OCT/IVUS provides endovascular information about the aetiology of the ischaemia (particularly in plaque disruption phenotypes) [5,7,12], while functional testing assesses endothelial/smooth muscle function [15,24]. During the aetiological investigation phase of the acute episode, proposed treatment corresponds to the suspected mechanism. More specifically, as per AHA guidelines, a different specific regime should be given to patients with plaque disruption, coronary spasm, microvascular dysfunction, embolism/thrombus and dissection, all of which have OCT/IVUS or functional testing proposed in addition to coronary angiography as diagnostic investigations, suggesting the increasing acceptance of their utility in aetiological/physiology assessment (CMR was listed as a determinant of non-ischaemic aetiology) [2].

More specifically, initial management of patients with a working diagnosis of MINOCA is distinguished by the AHA from a pathophysiological perspective, differentiating between ischaemic/true MINOCA subtypes and non-ischaemic mimicking causes; novel imaging modalities have already been proposed to contribute to this process. Plaque disruption, ideally investigated by intracoronary imaging (IVUS/OCT), should be treated with aspirin; high-intensity statins; β-blockers; ACE inhibitors/ARBs; and, possibly, clopidogrel or ticagrelor. Coronary spasms, in which a provocative spasm test can play a role, among other tests, can intuitively be treated with CCBs or other antispastic agents (nitrates, nicorandil, or cilostazol) with the possible addition of a statin. Microvascular dysfunction, as investigated by angiographic review and microvascular functional testing, might be successfully managed with antianginal therapies (CCBs; β-blockers; or unconventional regimens including ranolazine, aminophylline etc.), while embolism/thrombus, where intravascular imaging can provide significant information, should be treated with antiplatelet or anticoagulant therapy or other specific therapies against hypercoagulable conditions. SCAD can also be identified via IVUS/OCT, as previously described, and patients could benefit from a combination of aspirin; β-blockers; and, possibly, clopidogrel. Supply–demand mismatch should be individually assessed, and its underlying condition should be treated. As for conditions mimicking MINOCA, branch “flush occlusion” or severe branch stenosis could potentially be identified by intravascular imaging targeting plaque pathology (in addition to angiography or echocardiography screening for thrombi, endocarditis, and PFO) and should be managed with antiplatelets/anticoagulants, statins, β-blockers and ACEi/ARBs in the presence of LV dysfunction and possibly preserved EF. In this case, SCAD can be treated similarly to MINOCA-causing SCAD. Takotsubo syndrome, as previously stated, can be diagnosed using CMR, in addition to an LV angiogram, which would reveal the different types of ventricular ballooning, and can be treated with ACEi, possibly β-blockers and medical devices assisting in the case of heart failure. CMR is also of great utility in the diagnosis of cardiomyopathies and myocarditis, conditions in which medical or device therapies play a role in managing heart failure/LV dysfunction; myocarditis may also call for immunosuppressive or immunomodulatory therapies. The above represent, in large part, empirical/selective treatments, warranting further mechanism-specific management research [2]. Stenting of non-obstructive culprit lesions remains controversial. Prati et al. used OCT to detect culprit plaque erosions, later comparing percutaneous coronary intervention (PCI) to DAPT treatment. They found that all patients remained asymptomatic at 753 days [36], a finding confirmed by the EROSION study [37]. In a different OCT study, Imola et al. assessed stenting in lesions of unclear significance, finding no deaths, MIs or stent thrombosis in 4.6 ± 3.2 months. Despite these results, stenting in MINOCA remains a not fully investigated subject, and its utility remains to be determined. Specific working groups advise against the stenting of MINOCA lesions. It has also been stated that PCI should not be performed in patients with vasospastic angina, although it constitutes a viable option when this condition does not respond to medication [38]. Of note, although PCI is not routinely performed in patients where plaque disruption is the identified aetiology, targeted treatment with aspirin and statins is strongly recommended. In SCAD, despite stenting carrying the possibility of dissection exacerbation, in acute lumen obstruction due to intramural haematoma, a cutting balloon used to fenestrate and release the haematoma may prove to be beneficial [39].

Secondary prevention stands out as a prime candidate to be positively affected by mechanism-driven diagnosis, along with MINOCA treatment at the time of the acute index episode, areas significantly less advanced compared to CAD-Mis [40]. It is also what remains largely uninvestigated, rendering a review of the literature useful. In the HARP-MINOCA study, the authors stated that the identification of plaque disruption in patients with myocardial infarction and non-obstructive coronary artery disease carries important therapeutic implications, as such individuals are likely to benefit from antiplatelet therapy and statins. Nevertheless, such patients are less frequently prescribed standard secondary prevention treatments, including aspirin, clopidogrel, and statins. Despite the absence of obstructive disease, they face an approximately 2% risk of death or reinfarction within 6–12 months and a 15% rate of readmission within 6 months. Women with plaque disruption may represent a particularly high-risk subgroup, especially when appropriate secondary prevention is not implemented. Although this remains a hypothesis, further studies incorporating intravascular ultrasound during angiography, along with longitudinal outcome assessment, could help clarify this association [4,12]. In a study by Gerbaud et al., CMR-determined MINOCA resulted in a change in therapy in 16% of patients, while OCT resulted a change in therapy in 27.5% of patients [22]. The secondary prevention evidence in MINOCA can be synthesised around three main questions—the benefit of statins and ACEi/ARBs, the role of DAPT, and the position of these findings relative to current guidelines—each of which is addressed in turn.

Regarding statins and ACEi/ARBs, the evidence is consistent across independent datasets. In a large observational study of 9466 patients from the SWEDEHEART registry, Lindahl et al. demonstrated that statin therapy and ACEi/ARB use were each independently associated with reduced recurrent MACE, with β-blockers showing a trend toward benefit [35]. These findings align with the OCT-based review by Tani et al., which reported statin-associated MACE reductions of approximately 23% and ACEi/ARB-associated reductions of approximately 18% in MINOCA patients [11], and are further supported by the meta-analysis of Borzillo et al. [5]. The convergence of a large registry, an imaging-informed review, and a meta-analysis across different patient populations and study designs provides substantive, albeit observational, support for anti-atherosclerotic therapy in MINOCA. Whether this benefit extends equally across all MINOCA aetiologies—including vasospastic and microvascular subtypes—or is primarily driven by the plaque disruption subgroup remains uncertain, as most included studies did not perform systematic intracoronary imaging to stratify patients by mechanism.

The position of these findings relative to AHA guideline recommendations warrants clarification. The 2019 AHA Scientific Statement notes that the atherosclerotic burden in MINOCA is lower than in MI-CAD and, accordingly, does not issue a strong class I recommendation for statins in all MINOCA patients [2]. This reflects the absence of dedicated RCT evidence at the time of publication, not a conclusion that statins are ineffective; the guideline explicitly acknowledges that present data are insufficient to make firm recommendations. Therefore, the accumulating registry and observational data reviewed here do not contradict the guideline but, rather, represent the evolving evidence base that future guideline revisions will need to incorporate. The MINOCA BAT trial—designed to randomise at least 3500 patients to ACEi/ARBs and β-blockers or placebo, with all-cause mortality and cardiovascular events as endpoints—is expected to provide the RCT-level evidence that is currently absent [2].

The question of DAPT is the most complex, and the apparent contradiction between studies is explained by differences in patient selection and intracoronary imaging utilisation. In the SWEDEHEART registry and the OCT-based review by Tani et al., DAPT was not associated with a significant reduction in MACE in unselected MINOCA populations [11,35]. By contrast, the meta-analysis by Borzillo et al. identified a survival benefit associated with DAPT [5]; however, the authors, themselves, acknowledge that intracoronary imaging rates in the included studies were low, meaning that MINOCA patients with plaque disruption—the subgroup with the clearest biological rationale for antiplatelet therapy—were not systematically identified and could not be separately analysed. It is therefore likely that DAPT benefit in the Borzillo meta-analysis was driven by the subset of patients with unrecognised plaque disruption, which was diluted within a mixed MINOCA population. This interpretation is mechanistically coherent: antiplatelet therapy is most rationally indicated in plaque rupture and erosion, where thrombus formation is the culprit mechanism and is unlikely to be beneficial—and may be unnecessary—in vasospastic or microvascular MINOCA. Conversely, patients with cardioembolic MINOCA may derive greater benefit from anticoagulation than from antiplatelet therapy [5]. Therefore, the role of antiplatelet therapy in MINOCA is not globally established or refuted; it is mechanism-dependent, and the low intracoronary imaging rates in most pharmacotherapy studies remain the critical methodological limitation preventing definitive conclusions [41]. The ongoing MINOCA BAT trial is designed to randomize at least 3500 patients with MINOCA to receive either ACE inhibitors/ARBs and β-blockers or a matching placebo. It will assess all-cause mortality and cardiovascular events at 1 year, with the aim of clarifying the benefits of routine cardioprotective therapy in this population. Importantly, in MINOCA patients with evidence of atherosclerosis, modifiable coronary artery disease risk factors such as smoking, hypertension, diabetes mellitus, and hyperlipidaemia should be managed aggressively [2].

## 8. Current Limitations

Despite the rising use of the discussed relatively new diagnostic imaging techniques, patient outcomes seem far from being optimized. In their study, Gerbaud et al. mention that even though OCT and CMR improve clinical diagnosis and allow for appropriate clinically proven therapeutic interventions, in addition to providing a definite diagnosis to the patient, may also increase compliance with recommended therapies, whether CMR coupled with OCT-guided therapy can improve clinical outcomes in patients with MINOCA must be investigated in dedicated studies [22]. The systematic review and meta-analysis by Mileva et al. point out that current studies on MINOCA prognosis include patients with miscellaneous non-ischaemic pathologies and, therefore, the estimated prognoses of these patients vary because the prescribed drugs are not targeted to them correctly [19]. Most importantly, they reinforced the fact that no RCT has shown that secondary prevention drugs have improved the prognosis of these patients, as is true. Tani et al. suggested the potential administration of antiplatelet therapy based on the OCT-detected culprit lesion; however, such a practice has not yet been introduced formally as a norm [11]. Borzillo et al. noted that dual antiplatelet therapy (DAPT) has been associated with a survival benefit in patients with MINOCA, while angiotensin-converting enzyme inhibitors and angiotensin receptor blockers are linked to reductions in major adverse cardiac events, as suggested by a meta-analysis of their group [5]. However, the interpretation of these findings is limited by the low use of intracoronary imaging in the included studies. It is likely that the benefit of DAPT is confined to patients with plaque disruption, such as erosion or rupture. In contrast, patients with cardioembolic causes may be more appropriately managed with anticoagulation, whereas those with vasospastic mechanisms are more likely to benefit from vasodilator therapy. They conclude that intracoronary imaging leading to tailored therapy among MINOCA patients, along with the cost-effectiveness of such approach in a prospective study, would be beneficial; however, clinical data comparing the two are, so far, limited, a factor limiting this practice from becoming the standard [5]. Lastly, functional testing assessing the cardiac microvasculature may not always provide a definite diagnosis, even in cases where true microvascular dysfunction is present, since pharmacological stimulation with acetylcholine, which is commonly used to assess epicardial vasomotor abnormalities, can also induce microvascular smooth muscle constriction, making it difficult to attribute coronary microvascular dysfunction to a single mechanism, given the frequent overlap of underlying causes [42]. Therefore, new imaging modalities have yet to demonstrate the improvements in clinical outcomes that their diagnostic capabilities would suggest. This can, in major part, be attributed to a lack of randomized clinical trials; a consensus with respect to the meaning of each specific finding in imaging of MINOCA patients; and, lastly, the so far often undetermined pathophysiology of the acute clinical syndrome of each patient.

## 9. Current Insights and Future Directions

As demonstrated by the several different studies mentioned, new imaging techniques have a pivotal role in diagnosis, prognosis and treatment decision-making. Their insights in accurate detection of MINOCA against non-ischaemic conditions and pathophysiology present a unique opportunity to improve the clinical outcomes of MINOCA patients. Based on this reasoning, the PROMISE trial was the first randomized controlled trial designed to assess the effectiveness of incorporating advanced diagnostics into personalized treatment based on risk stratification. Patients with MINOCA were randomized 1:1 to either a stratified treatment based on a comprehensive diagnostic workup aimed at identifying the underlying aetiology or to standard care. The trial assessed the intergroup difference in angina status at 12 months, along with MACE frequency. More specifically, 101 patients with suspected MINOCA were randomized 1:1 to either a stratified treatment regimen based on the mechanism of the acute index event as detected by advanced diagnostic techniques or the standard treatment for MI; of these, 92 were confirmed as MINOCA and included in the final analysis (mean age: 62 ± 13 years; 48% women), with 45 patients in the stratified treatment arm and 47 in the standard of care arm. In particular, the comprehensive diagnostic workup led to reclassification of the initially suspected aetiology in 75.5% of cases, underscoring why MINOCA should be approached as a working diagnosis rather than a definitive diagnosis. The intervention-group patients underwent OCT if plaque disruption was suspected, ACh testing for vasomotor disorders, CMR, and transoesophageal echocardiography with or without contrast if thromboembolism was suspected in the presence of risk factors. According to each finding, patients with unstable plaque received DAPT ± PCI and statins, SCAD patients received SAPT or DAPT ± PCI and β-blockers, epicardial or microvascular spasm patients were administered CCBs, and patients with embolisms were anticoagulated. Patients with an undetermined aetiology received β-blockers, statins, antithrombotic therapy, and ACEi/ARNi. In cases where the causative mechanism was not identified, patients were managed with an empirical regimen that included antithrombotic therapy tailored based on clinical judgment (SAPT, DAPT, or oral anticoagulation if indicated), beta-blockers and RAAS inhibitors for their cardioprotective and anti-ischaemic properties (if tolerated), and statins targeting potential underlying atherosclerotic disease or endothelial dysfunction. In the standard-of-care group, patients received no additional workup besides CMR in selected cases, and their treatment consisted of SAPT or DAPT, along with other traditional, guideline-driven prescriptions. Patients with non-MINOCA diagnoses were excluded. At 12 months, patients in the stratified treatment group had an SAQSS (Seattle Angina Questionnaire Summary Score) of 82.7 ± 7.3, compared to the 74.7 ± 10.3 of the standard-of-care group (*p* < 0.001), a relative superiority shown across physical limitation, angina stability, angina frequency, treatment satisfaction, and quality of life, parameters that the questionnaire examined. Additionally, MACE occurred in five patients, with no significant difference between the two study groups [4 (8.5%) in the standard-of-care group vs. 1 (2.2%), *p* = 0.186]; nevertheless, we should note the low count of events [43].

The PROMISE trial is the first randomized trial recognizing the utility of advanced diagnostic techniques in clinical outcome. However, there are several limitations of the PROMISE trial that should be acknowledged. More specifically, the trial enrolled only 101 patients (92 analysed), was not blinded, had a short follow-up of 12 months, and used a patient-reported outcome (SAQ summary score) as the primary endpoint rather than hard clinical events; furthermore, the MACE comparison was clearly underpowered (1 vs. 4 events, *p* = 0.186). MINOCA has evolved from purely a diagnosis of exclusion to a mechanism-based syndrome, which makes the opportunity for physiology-based treatment evident. Thus, it is reasonable to recommend that clinicians consider employing these imaging modalities when working on a diagnosis of MINOCA, utilizing the prognostic and treatment decision-making data each has shown to offer.

**Figure 2:**
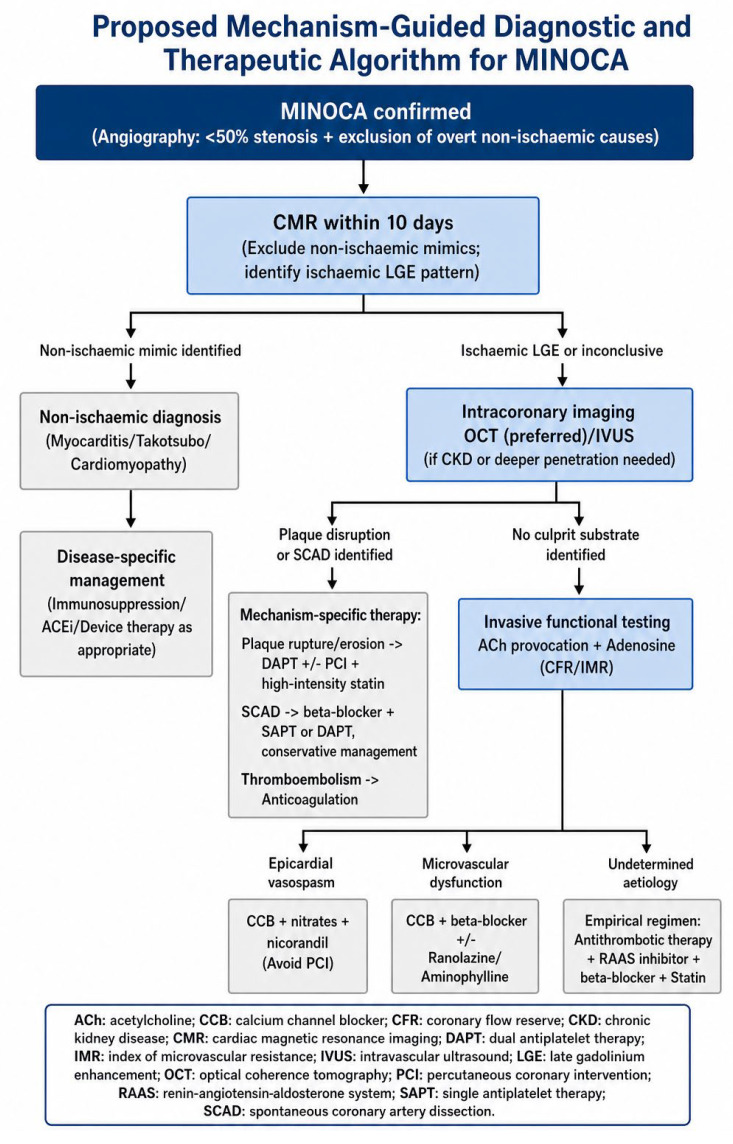
Proposed mechanism-guided diagnostic and therapeutic algorithm for MINOCA.

Looking beyond current practice, artificial intelligence (AI)-assisted image analysis represents a promising frontier in MINOCA diagnostics. In CMR, deep learning algorithms are being developed for automated LGE segmentation and pattern classification—distinguishing ischaemic subendocardial/transmural enhancement from non-ischaemic subepicardial/intramyocardial patterns—with early studies reporting performance comparable to that of experienced readers [44]. In OCT, AI-based automated plaque characterisation, including fibrous cap thickness measurement and thrombus detection, has demonstrated high accuracy against expert annotation, with the potential to reduce operator-dependent variability, which currently limits reproducibility [45]. Integration of multimodal imaging data—combining CMR tissue characterisation, the OCT-derived plaque phenotype, and functional testing haemodynamics—into machine learning-based risk stratification models may ultimately enable precision outcome prediction beyond what any single modality achieves independently. However, prospective validation of AI-derived MINOCA phenotypes in dedicated outcome trials does not yet exist, and the clinical adoption of these tools remains at an early stage. Their inclusion in future MINOCA trial design, both as diagnostic aids and as endpoints for protocol standardisation, represents an important and underexplored opportunity (Figure 2).

## 10. Conclusions

In conclusion, MINOCA is no longer the diagnostic dead end it was once considered in the past. Cardiac magnetic resonance is the non-invasive reference standard for distinguishing true ischaemic MINOCA from non-ischaemic mimics, with reclassification rates that meaningfully alter both prognosis and management. Intracoronary imaging, with mainly OCT and complementarily IVUS, exposes the culprit vascular substrate at a resolution unavailable to angiography, while invasive functional testing with acetylcholine and adenosine extends the diagnostic reach to vasomotor and microvascular pathology, separating endothelium-dependent from endothelium-independent dysfunction. Until the evidence matures, the available data already support a clear paradigm shift: in any patient presenting with suspected MINOCA, the initial label should be regarded as provisional, and a multimodal diagnostic strategy combining CMR with intracoronary and/or functional assessment should be pursued whenever feasible. Such an approach refines the diagnosis; sharpens prognostication; and, most importantly, opens the door to personalised, mechanism-based therapy, which remains the most promising route to reducing the still substantial morbidity and mortality of this once-overlooked condition.

## Figures and Tables

**Table 1:** Summary of the diagnostic examinations used for MINOCA diagnosis.

Parameter	Cardiac Magnetic Resonance (CMR)	Optical Coherence Tomography (OCT)	Intravascular Ultrasound (IVUS)	Functional Testing (ACh/Adenosine)
Invasiveness	• Non-invasive • No ionising radiation	• Invasive (intracoronary catheter) • Requires contrast flush (low volume) • Performed during index catheterisation	• Invasive (intracoronary catheter) • No contrast required • Performed during index catheterisation	• Invasive (intracoronary infusion) • ACh: endothelium-dependent testing • Adenosine: endothelium-independent testing
Optimal timing	• Within 10 days of acute event (transient oedema on T2WI may normalise after 10–14 days)	• At index catheterisation • Thrombus may resolve if delayed	• At index catheterisation • Plaque features stable over time	• Index or elective catheterisation • Vasospasm testing safe beyond acute phase
Identified mechanisms	• True ischaemic MINOCA (subendocardial/transmural LGE) • Myocarditis (subepicardial/intramyocardial LGE) • Takotsubo (absent LGE, characteristic wall motion) • Non-ischaemic cardiomyopathies	• Plaque rupture and erosion • Intraplaque haemorrhage and layered plaque • Intramural haematoma; SCAD • Intimal bumping/wavy intima (vasospasm) • Thrombus characterisation	• Plaque rupture, ulceration, and calcification • Intramural haematoma; SCAD • Lipid core assessment (NIRS-IVUS) • Plaque burden in non-obstructive segments	• Epicardial vasospasm: ≥90% constriction + symptoms (ACh) • Microvascular spasm: symptoms without epicardial narrowing (ACh) • Microvascular dysfunction: reduced CFR and elevated IMR (adenosine)
Therapeutic impact	• Reclassifies non-ischaemic mimics → avoids unnecessary antiplatelet/statin therapy • Management changed in a minority of patients	• Management changed in 27.5% of patients • Identifies plaque disruption phenotype → antiplatelet + statin (±PCI for erosion) • Distinguishes SCAD → β-blocker; conservative management	• Guides need for antiplatelet/statin in non-obstructive plaque • Identifies SCAD in angiographically normal segments • Preferred over OCT in CKD	• Vasospasm → CCBs, nitrates, and nicorandil • Microvascular dysfunction → CCBs, β-blockers, ranolazine, and aminophylline • Guides avoidance of PCI in vasospastic angina
Key limitations	• Does not visualise culprit vascular lesion • Transient oedema normalises after ~14 days • Gadolinium contraindicated in severe CKD • Availability and cost limit routine use	• Limited tissue penetration (1–2 mm) • Pre-procedure nitroglycerine may mask vasospasm • Operator-dependent image acquisition and interpretation	• Lower resolution than OCT (axial ~100 μm vs. ~15 μm) • Cannot characterise thrombus or intimal detail as precisely as OCT • Operator-dependent; less widely available in acute setting	• Pharmacological stimulation may simultaneously activate epicardial and microvascular smooth muscle, complicating attribution • Not standardised across centres (ACh dose/protocol varies) • Hard outcome data in MINOCA remain limited

## Data Availability

The raw data supporting the conclusions of this article will be made available by the authors upon request.

## Abbreviations

ACEi	Angiotensin-Converting Enzyme Inhibitor
ACh	Acetylcholine
ACS	Acute Coronary Syndrome
AHA	American Heart Association
AMI	Acute Myocardial Infarction
ARB	Angiotensin Receptor Blocker
ARNi	Angiotensin Receptor-Neprilysin Inhibitor
CAD	Coronary Artery Disease
CCB	Calcium Channel Blocker
CFR	Coronary Flow Reserve
CI	Confidence Interval
CKD	Chronic Kidney Disease
CMR	Cardiac Magnetic Resonance Imaging
DAPT	Dual Antiplatelet Therapy
ECG	Electrocardiogram
EF	Ejection Fraction
HR	Hazard Ratio
IMR	Index of Microvascular Resistance
IQR	Interquartile Range
IVUS	Intravascular Ultrasound
LGE	Late Gadolinium Enhancement
LMCA	Left Main Coronary Artery
LV	Left Ventricle/Left Ventricular
LVEF	Left Ventricular Ejection Fraction
MACE	Major Adverse Cardiovascular Events
MACCE	Major Adverse Cardiac and Cerebrovascular Events
MI	Myocardial Infarction
MI-CAD	Myocardial Infarction with Coronary Artery Disease
MINOCA	Myocardial Infarction with Non-Obstructive Coronary Arteries
NICM	Non-Ischaemic Cardiomyopathy
NIRS	Near-Infrared Spectroscopy
NO	Nitric Oxide
OCT	Optical Coherence Tomography
OR	Odds Ratio
PCI	Percutaneous Coronary Intervention
PFO	Patent Foramen Ovale
RAAS	Renin-Angiotensin-Aldosterone System
RCT	Randomized Controlled Trial
SAQ	Seattle Angina Questionnaire
SAPT	Single Antiplatelet Therapy
SAQSS	Seattle Angina Questionnaire Summary Score
SCAD	Spontaneous Coronary Artery Dissection
STEMI	ST-Elevation Myocardial Infarction
STIR-T2	Short TI Inversion Recovery T2-Weighted Imaging
TLR	Target Lesion Revascularization
